# Turner syndrome with isochromosome Xq as a cause of granulomatous hepatitis: a case report

**DOI:** 10.1097/MS9.0000000000002042

**Published:** 2024-04-15

**Authors:** Ayman F. Ali, Lina Haffar

**Affiliations:** Faculty of Medicine, Damascus University, Damascus, Syria

**Keywords:** case report, granulomatous hepatitis, hepatic granuloma, isochromosome xq, Turner syndrome, UDCA

## Abstract

**Introduction:**

Turner syndrome (TS) is the most common sex chromosome abnormality in women, caused by a complete or partial absence of the second sex chromosome. The karyotype 46, X,i(Xq) is the underlying cause in about 10% of the cases of TS. Hepatic abnormalities are frequent in TS. Granulomas are relatively common in liver samples but are very rarely reported in TS.

**Case presentation:**

A 15-year-old female with TS attended a consultation for evaluation of elevated liver enzymes. Her chromosomal analysis showed mosaicism 46, X (iso xq)100%. There were no stigmata of chronic liver disease. A liver biopsy showed granulomatous hepatitis. Other causes of hepatic granulomas have been excluded. Ursodeoxycholic acid (UDCA) therapy leads to the normalization of transaminases.

**Clinical discussion:**

Although Hepatic involvement is common and mostly asymptomatic in TS, the mechanism of liver injury is not well understood. The hepatic histological changes in these cases are variable and range from minimal abnormalities to nonalcoholic steatohepatitis (NASH), liver architectural changes, and biliary lesions. Hepatic granulomas are associated with a wide range of systemic disorders but are very rarely reported in tuner syndrome. Normalization of liver enzymes after treatment with UDCA was previously reported, but the importance of this approach is to be determined.

**Conclusion:**

Granulomatous hepatitis may be associated with TS and may be added to the histological patterns encountered in this disorder.

## Introduction

Highlights

- Patients with Turner syndrome commonly demonstrate abnormal liver function tests.
- The liver involvement is mostly asymptomatic.
- The pattern of hepatic histological changes ranges from minimal abnormalities to nonalcoholic steatohepatitis (NASH), architecture changes, and biliary lesions.
- Hepatic granulomas are unusual in Turner syndrome and could be part of wide spectrum of histological changes in this disorder.
- Ursodeoxycholic acid (UDCA) may be beneficial in normalizing liver enzymes, but there is no data about its long-term use.

Turner syndrome (TS) is a rare genetic condition with multisystem involvement caused by absence or insufficiency of the second sex chromosome. Half of the affected girls have 45, X monosomy while the remainder have mosaicism or structural anomalies of the X chromosome^1^. Genotype 46, X,i(Xq) is the most prevalent among the structural X chromosome abnormalities, accounting for 10% of cases^2^.

Liver function test (LFT) abnormalities are frequent in TS (20–80%)^3^ making follow-up and referral to a hepatologist necessary. The published guidelines suggest screening labs to assess liver function starting in the school-aged years^4^. The mechanism of liver disease in TS is not well understood, and liver biopsies have not consistently defined any unique or diagnostic pathology^5^. Three main types of pathological changes have been described: steatohepatitis, vascular damage, mostly observed in regenerative nodular hyperplasia (RNH), and autoimmune disease, such as primary biliary cholangitis^5,6^.

Hepatic granulomas are relatively common in liver samples, associated with a variety of systemic conditions, or may be an incidental finding on an otherwise normal liver biopsy^7^. There is only one report of the association of TS with hepatic granulomas^8^. Here, we describe a case of TS associated with hepatic granulomas and the effects of ursodeoxycholic acid (UDCA) treatment on liver tests. This article has been reported in line with the SCARE 2023 criteria^9^.

## Case presentation

**Figure 1:**
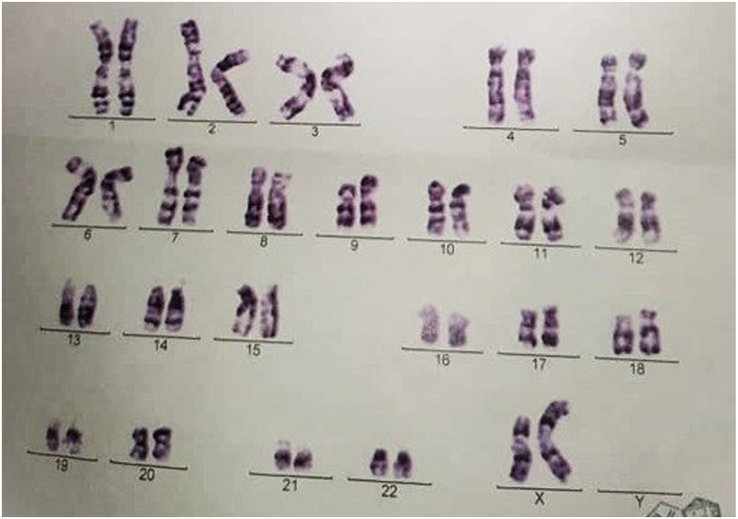
Karyotyping from peripheral blood shows female: 46 X (iso xq) 100%.

We report on a 15-year-old female with TS who attended a consultation for evaluation of elevated liver enzymes. She had hypothyroidism, short stature, and secondary amenorrhoea. Her chromosomal analysis showed mosaicism 46, X (iso xq)100% (Fig. 1).

She had abnormal LFTs for 4 years and was referred to our Hepatology clinic for evaluation before starting hormone replacement therapy. The Patient was the fourth child of consanguineous parents. Her menstruation spontaneously started when she was 13 years old and ceased after three cycles. Her past medical history, including antenatal and postnatal periods, was uneventful. Aside from amenorrhoea and short stature, she was asymptomatic and had no significant medical problems. There was no family history of liver or genetic disorders. At the time of presentation, she was not taking any medication. Her past medication history was not significant. Physical examination showed a short-statured female without any stigmata of end-stage liver disease. The abdominal ultrasound was unremarkable. Laboratory data is shown in Table 1.

**Table 1:** Laboratory data.

Laboratory test		Normal
Alanine transaminase (ALT)	423 U/l	5–45 U/l
Aspartate transaminase (AST)	324 U/l	5–45 U/l
Alkaline phosphatase (ALP)	220 U/l	Up to 258 U/l
Total bilirubin/direct bilirubin	0.53/0.27 mg/dl	Up to 1 mg/dl
Albumin	4.1 mg/dl	3.4–5.4 mg/dl
PT/INR	100%/1	INR <1.1
Hepatitis: B, C panel	Negative	Negative
ANA	Negative	Negative
ASMA	Negative	Negative
Anti-LKM1	Negative	Negative
AMA	Negative	Negative
Ceruloplasmin	25 mg/dl	20–60
Anti-TTG-IgA	5.1 au/ml	0–18
Anti-deamidated gliadin peptide (DGP)	10.5	Negative <20 U/ml

AMA, Antimitochondrial Antibodies; ANA, Antinuclear Antibodies; ASMA, Anti-smooth Muscle Amtibodies; PT/INR, Prothrombin Time/International normalised ratio.

**Figure 2:**
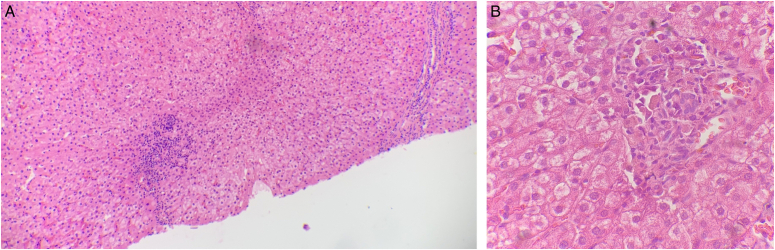
Liver histology. (A) Chronic inflammation in the portal space (low power), (B) hepatic granuloma (high power).

A percutaneous liver biopsy showed preserved hepatic architecture, mild lymphocytic infiltrate in the hepatic parenchyma, and focal granulomas in portal space consistent with granulomatous hepatitis (Fig. 2).

She had no respiratory symptoms and no lymphadenopathy. Her chest X-ray and ACE were within normal limits. Tuberculin skin test (TST) was negative.

We treated the patient with UDCA 250 mg t.i.d. After 1 month alanine transaminase (ALT) and aspartate transaminase (AST) were within normal limits. As there are no studies on the long-term use of UDCA in TS without obvious biliary lesions, we decided, after a discussion with the patient’s parents and her endocrinologist, to discontinue UDCA and start hormone replacement therapy.

## Discussion

We present a case of a rare variant of TS reported in a 15-year-old girl presenting with elevated liver enzymes. The patient’s karyotype analysis revealed the presence of X isochromosome-X syndrome [46 XXi(Xq)]

TS is the most frequently reported sex chromosomal abnormality, affecting ~1/2500 female newborns. Approximately 50% of individuals diagnosed with TS are identified as having the characteristic classic form, where there is a complete absence of one of the sex chromosomes resulting in a karyotype of 45, XO. In ~25% of TS cases, there are mosaic forms present. The remaining cases encompass structural abnormalities of the X chromosome. Isochromosome Xq, like in our case, is the most prevalent among the structural X chromosome abnormalities^1^ and is the underlying cause in 10% of the cases of TS^10^.

Hepatic abnormalities in patients with TS have been documented in the medical literature for over six decades, with the earliest report dating back to 1959 when Bridwell described portal fibrosis in a TS patient^11^. It is worth noting that liver function test abnormalities are quite common in individuals with TS, with a prevalence ranging from 20 to 80%, and these abnormalities tend to worsen as individuals age. Specifically, markers such as alkaline phosphatase, alanine/aspartate aminotransferase, and G-Glutamyl transferase (ALT, AST, GGT) often show elevated levels^3^.

A study conducted by Calanchini *et al.* ^12^ on a cohort of 125 women with TS revealed a correlation between raised liver function tests and a specific karyotype iso[X]q. Interestingly, another study conducted on the same subject matter yielded similar findings^13^. The existing body of evidence suggests that these elevations in liver function tests are generally benign and do not progress clinically or histologically over time^14–16^. However, some cases of advanced liver diseases and cirrhosis have been reported. A study conducted on a smaller cohort^6^ provided additional data on the progression and outcome of liver involvement in TS, demonstrating that 10 out of the 27 women with persistent liver enzyme elevation who underwent a liver biopsy showed architectural changes in their liver.

Liver biopsies conducted on adults with typical enzyme abnormalities have not consistently revealed any distinct pathology in women with TS^5^. Despite this, certain hepatic conditions have historically been more prevalent in individuals with TS, including nonalcoholic steatohepatitis (NASH), architectural changes in the liver, and biliary lesions.

Hepatic granulomas have been reported to occur in ~2–15% of liver biopsy specimens obtained from adults, and in around 4–7% of specimens from children^17,18^. Granulomas in the liver are often indicative of an underlying systemic process^19^, although can be an incidental finding on an otherwise normal liver biopsy, as in biopsies performed in prospective organ donors^7^, or can be a manifestation of a disorder primarily of the liver. It is important to emphasize that the granulomas rarely cause structural damage to the liver.

The previous literature has rarely reported a link between TS and the presence of a granulomatous process^8,20^, suggesting that this could be attributed to the autoimmune disorders frequently observed in individuals with TS. Our patient has hypothyroidism, which is of autoimmune nature. Sarcoidosis and tuberculosis have been ruled out.

After excluding other potential causes of hepatic granulomas, the patient was initiated on treatment with UDCA, which led to the normalization of liver enzyme levels after 1 month. The exact mechanism by which UDCA exerts its beneficial effects in this case remains unclear. It is known that UDCA possesses several properties, including cytoprotective, immunomodulatory, antioxidative, and anti-inflammatory effects^21^. One study^6^ suggested that the use of UDCA in TS with cholestatic profile of liver test alterations or intrahepatic bile duct changes has limited impact on the histologic characteristics of the liver, despite occasional improvements in liver biochemistry. In our case, normalization of liver tests has been observed despite the absence of any serological or histological markers of cholestasis.

This case demonstrates the wide range of histopathological changes of the liver encountered in TS, but certain aspects, including the correlation between the karyotype and liver injury patterns, as well as the impact of long-term treatment with UDCA, require further investigation in future controlled studies.

## Conclusion

LFT abnormalities are commonly observed in patients with TS. Hepatic granulomas may be considered a possible cause of abnormal LFT in TS. UDCA may provide some beneficial effects, even in the absence of overt biliary lesions.

## Ethical approval

Ethical approval for this study was provided by the Ethical Committee of our Hospital (Al-Assad University Hospital, Damascus).

## Consent

Written informed consent was obtained from the patient's parents for publication and any accompanying images. A copy of the written consent is available for review by the Editor-in-Chief of this journal on request.

## Source of funding

None.

## Author contribution

A.A.: followed the patient, wrote the article. L.H.: made the histopathological diagnosis. All authors read and approved the final version of the manuscript.

## Conflicts of interest disclosure

The authors declared no conflicts of interest.

## Research registration unique identifying number (UIN)

NA as this is a case report.

## Guarantor

None.

## Data availability statement

All data generated or analyzed during this study are included in this article. Further inquiries can be directed to the corresponding author

## Provenance and peer review

None.

## Presentation

None.
